# Risk factors for subsequent lupus nephritis in patients with juvenile-onset systemic lupus erythematosus: a retrospective cohort study

**DOI:** 10.1186/s12969-023-00806-x

**Published:** 2023-03-24

**Authors:** Tzu-Chuan Hsu, Yao-Hsu Yang, Li-Chieh Wang, Jyh-Hong Lee, Hsin-Hui Yu, Yu-Tsan Lin, Ya-Chiao Hu, Bor-Luen Chiang

**Affiliations:** 1 Department of Paediatrics, National Taiwan University Hospital, No. 8 Chung-Shan South Road, Taipei, 100 Taipei, Taiwan; 2 Department of Paediatrics, Taipei Medical University Hospital, Taipei, Taiwan; 3 Department of Paediatrics, National Taiwan University Hospital Hsinchu Branch, Hsinchu, Taiwan; 4 Graduate Institute of Clinical Medicine, College of Medicine, National Taiwan University, Taipei, Taiwan

**Keywords:** Subsequent lupus nephritis, Juvenile-onset systemic lupus erythematosus, Anti-dsDNA antibody, Erythrocyte sedimentation rate

## Abstract

**Background:**

Lupus nephritis (LN) is a crucial organ involvement in systemic lupus erythematosus (SLE). Patients with LN have higher morbidity and mortality rates than those without. Among all patients with LN, 20–40% had delayed onset, but the data for patients with juvenile-onset SLE (jSLE), who have a higher percentage of LN than patients with adult-onset SLE (aSLE), were limited. This study aimed to determine the risk factors for subsequent LN in patients with jSLE.

**Methods:**

A retrospective cohort study was conducted between 2008 and 2018 in a single tertiary medical centre. Patients with diagnosed jSLE were reviewed. We investigated those without LN at diagnosis and whether they developed LN afterward. The primary outcome was the development of subsequent LN. Clinical manifestations at diagnosis, serial laboratory data, and treatments were reviewed during follow-up periods.

**Results:**

Among the 48 patients with jSLE without initial LN, 20 developed subsequent LN later (Group 1), whereas 28 remained free of LN (Group 2). There was no difference in the percentage of initial manifestations except for more discoid rashes in Group 2 patients. In the Cox regression model, elevated average anti-double-stranded DNA (dsDNA) antibody, low average serum complements, and high average erythrocyte sedimentation rate (ESR) levels during follow-up were predictors of subsequent LN. After adjusting for these factors in multivariable analyses, only high average anti-dsDNA antibody and high average ESR levels remained predictive of subsequent LN. For every 100 IU/ml increase in anti-dsDNA antibody, the risk for subsequent LN in jSLE increases by 1.29 times (hazard ratio = 1.29, 95% confidence interval 1.055–1.573).

**Conclusion:**

Persistently high anti-dsDNA antibody and ESR levels during the follow-up period were risk factors for subsequent LN in patients with jSLE.

**Supplementary Information:**

The online version contains supplementary material available at 10.1186/s12969-023-00806-x.

## Background

Systemic erythematous lupus (SLE) is characterised by a breach in self-tolerance and propagation of multiple autoantibodies, presenting with heterogeneous phenotypes and a wide range of organ involvement. Lupus nephritis (LN) is one of the most threatening vital organ involvements in patients with SLE, and those with renal involvement have a higher morbidity and mortality rate than those without LN [1–6]. The percentage of LN in SLE patients differs from race to race and tends to be higher in Asian patients with SLE than in Caucasians [7–12]. For those without LN initially, the time from SLE diagnosis to LN detection was often within 2 years. Tracing back to previous studies, the definition of delayed-onset LN or late-onset LN varied from more than 1 [13] to 5 years [14, 15] after SLE diagnosis, and several studies reported a poorer prognosis in those with delayed-onset LN than in those with LN at SLE diagnosis [13, 15, 16].

The prevalence of juvenile-onset systemic lupus erythematosus (jSLE) among Taiwanese patients is approximately 6.3 cases per 100,000 [17]. It is known that patients with jSLE are more prone to LN than those with adult-onset SLE (aSLE) [5, 18], and approximately 50% of patients with jSLE have renal involvement either at the time of SLE diagnosis (TD) or during their disease courses [19–24]. According to a large British cohort that enrolled 232 patients with jSLE, even up to 80% of them developed LN within 5 years of follow-ups [25]. Based on this idea, it is crucial to know the risk factors for subsequent LN in patients with jSLE. However, few studies have mentioned this issue in patients with jSLE.

In this study, we aimed to determine the risk factors associated with subsequent LN among patients with jSLE, as those with a higher risk of developing LN may benefit from more frequent follow-up that they can have early detection and treatment for LN.

## Methods

### Patients enrolment

Patients diagnosed with jSLE from 1 January 2008 to 31 December 2018 at the National Taiwan University Hospital (NTUH) were enrolled. The enrolled patients received regular follow-ups at the outpatient department every two to four weeks at diagnosis and then every one to three months afterward, which depended on each patient’s condition. All patients were diagnosed with SLE before their 18th birthday by fulfilling at least four of the 11 items of the American College of Rheumatology (ACR) 1997 criteria [26]. TD was defined as the time when a patient was diagnosed with SLE. All patients had at least two consecutive data with an interval of more than 1 month. Patients with other systemic autoimmune diseases or coexisting kidney diseases at baseline were excluded. Patients’ data were collected from the electronic medical records of NTUH.

Patients with jSLE who did not have LN at TD were classified into two groups. Group 1 patients had later LN development during follow-ups, defined as subsequent LN. The other patients who remained free of LN during follow-ups were classified into Group 2. LN was defined as persistent proteinuria (≥ 0.5 g per day, ≥ 3 + if quantification was not performed [27], or persistent urine total protein to creatinine ratio (UPCR) ≥ 0.5 [28]) plus the presence of RBC or WBC cellular casts in high-power field in urine analysis, or by histologically proven LN if a renal biopsy was performed.

### Study outcome and follow-up

The study population was followed up from baseline to the date patient was diagnosed of LN or until 22 July 2020. The primary endpoint was LN. The time-to-event was from the TD to the time when patients were diagnosed of LN. We reviewed the clinical characteristics, serial laboratory data at baseline and during follow-up, and therapeutic regimens of each patient. We recorded the initial haemogram at TD and serial levels of complements (C3, C4), erythrocyte sedimentation rate (ESR), and anti-dsDNA antibody during the follow-up period. The anti-dsDNA antibody levels were measured using the QUANTA Lite® dsDNA enzyme-linked immunosorbent assay (ELISA) kit from Inova Diagnostics, which only detects Immunoglobulin G (IgG). According to the manufacturer, the anti-dsDNA antibody level was positive once its value exceeded 300 IU/ml. For patients below 17 years of age, we use Bedside Schwartz Formula [29, 30] to measure their estimated glomerular filtration rate (eGFR). Furthermore, for patients who were 17 years old or older, their eGFR was calculated by a 4-variable MDRD equation [31].

Medications that had been taken for more than 1 month were analysed. Disease-modifying antirheumatic drugs (DMARDs) in our study included hydroxychloroquine (HCQ), methotrexate (MTX), cyclosporin (CsA), azathioprine (AZA), and mycophenolate mofetil (MMF). The accumulated corticosteroid dose for each patient was also calculated.

### Statistical analysis

Patient data were expressed as counts or percentages of ordinal data. For quantitative data, normally distributed variables were summarised using mean with standard deviation (SD), and non-normally distributed variables were summarised using median and range. The laboratory data collected during the follow-ups were analysed and presented as the arithmetic mean of all data to evaluate the average condition of this period. All analyses were conducted using IBM®SPSS® software (version 22.0; IBM Corporation, Armonk, NY, USA). T-tests and Mann–Whitney U tests were used for continuous variables. Chi-square and Fisher’s exact tests were used for categorical variables. Hazard ratios (HRs) and 95% confidence intervals (CI) values for LN development were analysed using Cox proportional hazard model and multivariable-adjusted proportional hazard models. All tests were two-sided, and a value of *P* < 0.05 was considered statistically significant.

## Results

### Patients’ characteristics at diagnosis and during follow-ups

**Fig. 1:**
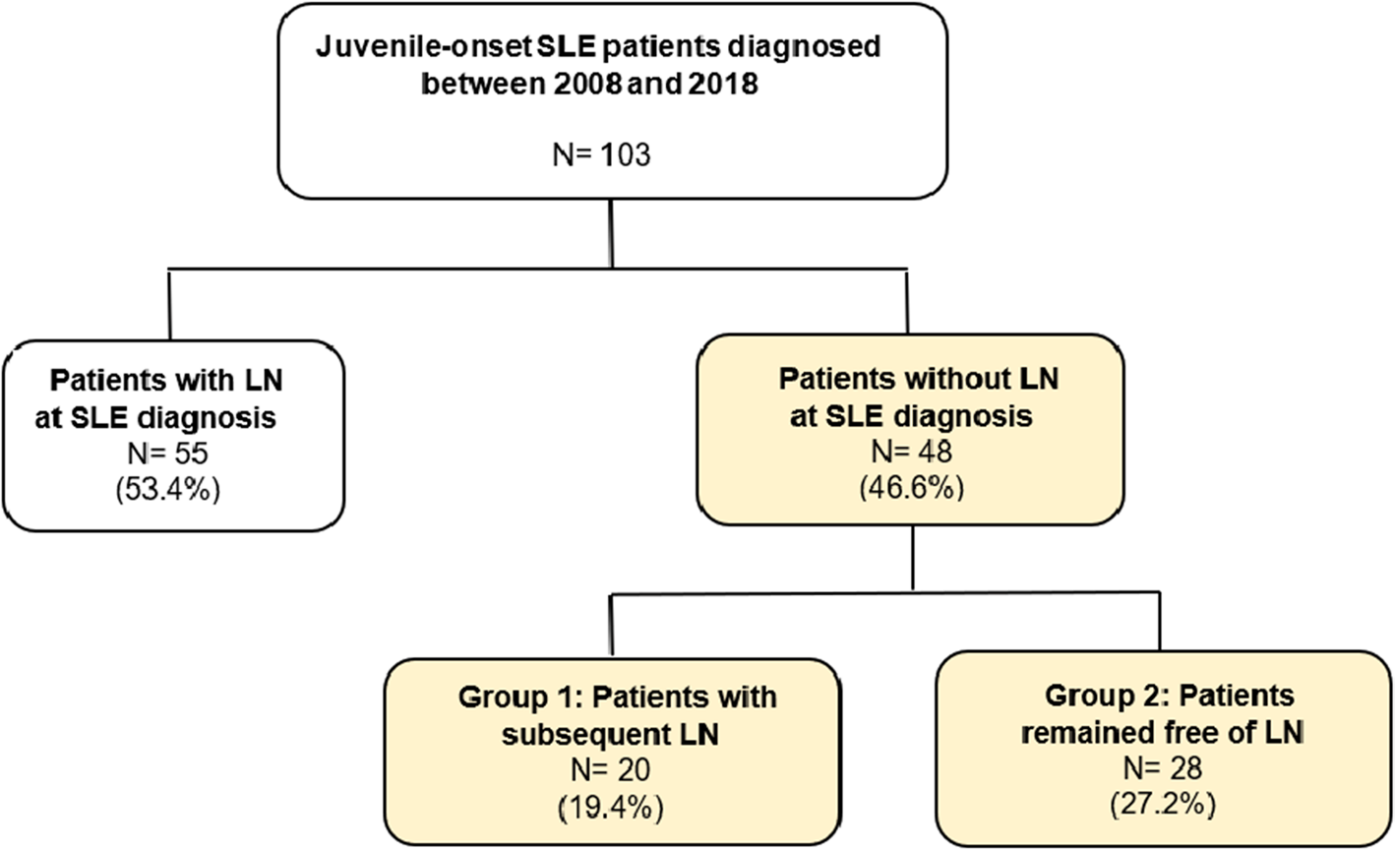
The algorithm of patient enrolment in the study. (SLE: systemic lupus erythematosus; LN: lupus nephritis)

The recruitment base for our study consisted of 103 jSLE patients (Fig. 1), and 55 patients (53.4%) had LN at TD or had LN within one month after TD. The other 48 patients who were free of LN were enrolled and being retrospectively reviewed. Twenty of the 48 patients had subsequent LN (Group 1), and the remaining 28 patients had no renal involvement until the last follow-up (Group 2). Table 1 shows the baseline characteristics of the two groups. Girls were the majority in both groups. The mean ages at TD in Group 1 and Group 2 patients were 13.1- and 13.2-year-old, respectively. The mean follow-up time was 4.6 and 5.8 years, and the shortest follow-up duration was 0.77 and 1.65 years in each group. In Group 1, the median time of LN development was 4.28 years, ranging from 0.77 to 12.1 years. A total of 12 patients (60%) developed subsequent LN within 5 years. The median age they developed LN was 17.9 years old, ranging from 11.9 to 26.8 years old.

**Table 1:** Demographic and clinical characteristics of patients with SLE at the time of diagnosis

	Total (*N* = 48)	Group 1 (*N* = 20)	Group 2 (*N* = 28)	*p*-value
Female gender, n (%)	42 (87.5)	18 (90.0)	24 (85.7)	1
Age at SLE diagnosis (years), mean (SD)	13.16 (2.4)	13.08 (2.1)	13.21(2.6)	0.855
Duration of follow-up (years), mean (SD)	5.32 (2.7)	4.63 (2.8)	5.82 (2.7)	0.140
Time to LN, median (range)		4.28 (0.8–12.1)	NA	
Age at LN diagnosis (years)		17.85 (11.9–26.8)	NA	
**Clinical manifestations (at SLE diagnosis), n (%)**				
Malar rash		12 (60.0)	15 (53.6)	0.771
Discoid rash		1 (5.0)	9 (32.1)	**0.031**
Photosensitivity		5 (25.0)	6 (21.4)	1
Oral ulcers		8 (40.0)	8 (28.6)	0.537
Arthritis		14 (70.0)	16 (57.1)	0.546
Serositis		4 (20.0)	3 (10.7)	0.429
Seizure/ psychosis		1 (5.0)	2 (7.1)	1
Hematologic		14 (70.0)	22 (78.6)	0.520
Immunologic		20 (100)	26 (92.9)	0.503
Antinuclear antibody		20 (100)	27 (96.4)	1
Hepatitis		3 (15.0)	5 (17.9)	1
**Hemogram (at SLE diagnosis), n (%)**				
Leukopenia^a^		3 (21.4)	9 (33.3)	0.494
Lymphopenia^b^		9 (64.3)	14 (51.9)	0.520
Thrombocytopenia^c^		3 (20)	5 (18.5)	1

Mean and SD (standard deviation) are presented for quantitative and ordinal data

^a^Leukopenia was defined as white blood cell count < 4000/ µL

^b^Lymphopenia was defined as absolute lymphocyte count < 1500/ µL

^c^Thrombocytopenia was defined as platelet count < 100,000/ µL

We compared the two groups in terms of clinical manifestations of TD and characteristics during follow-ups. No difference between the initial manifestations were noted, except for discoid rashes, which were less frequent in Group 1 (5.0% vs. 32.1%, *p* = 0.031). When comparing the laboratory data at TD, no differences between C3, C4, ESR, and anti-dsDNA status were noted in these two groups (Supplementary Table 1). Comparisons of the laboratory data and medications during the follow-up period are listed in Table 2. Anti-dsDNA antibody was ever positive (> 300 IU/ml) in 100% of Group 1 and 93% of Group 2 patients. The patients in Group 1 had a significantly higher mean of average anti-dsDNA antibody level than those in Group 2 (784.0 vs. 434.7 IU/ml, *p* < 0.0001). The mean of average C3 and C4 levels were significantly lower in patients with subsequent LN (mean of average C3: 66.9 vs. 86.5 mg/dl, *p* = 0.0007; mean of average C4: 10.1 vs. 14.0 mg/dl, *p* = 0.0047).

**Table 2:** Comparison of the laboratory data and medications during the follow-up time among the two groups

	Group 1 (*N* = 20)	Group 2 (*N* = 28)	*p*-value
**Presence of autoantibodies**			
anti-dsDNA Ab	20 (100)	26 (92.9)	0.504
anti-Smith Ab	12 (63.2)	10 (38.5)	0.136
anti-RNP Ab	11 (57.9)	11 (42.3)	0.373
anti-SSA Ab	13 (68.4)	20 (76.9)	0.734
anti-SSB Ab	7 (36.8)	13 (50.0)	0.545
Ever positive dRVVT	6 (35.3)	7 (28.0)	0.750
**Average laboratory tests during the follow-up period**			
anti-dsDNA (IU/mL), mean (SD)	784.0 (297.7)	434.7 (255.9)	**0.0001**
C3 (mg/dL), mean (SD)	66.9 (18.7)	86.5 (17.2)	**0.0007**
C4 (mg/dL), mean (SD)	10.1 (3.5)	14.0 (5.5)	**0.0047**
ESR (mm/hr), mean(SD)	31.84 (19.57)	22.61 (13.86)	0.0861
**Medication used during follow-up periods**^**a**^			
Accumulated steroid dose (mg/day), median (range)	6.2 (0.5–24.3)	5.1 (0–15.2)	0.085
HCQ	19 (95.0)	28 (100)	1
CsA	13 (65.0)	15 (53.6)	0.43
MTX	4 (20.0)	2 (7.1)	0.15
MMF	8 (40.0)	9 (32.1)	0.57
AZA	9 (45.0)	12 (42.9)	0.88

Values are n (%) unless otherwise specified

*SD* Standard deviation, *dsDNA* Double strand DNA, *Ab* Antibody, *dRVVT* Diluted Russell’s viper venom test, *ESR* Erythrocyte sedimentation rate, *HCQ* Hydroxychloroquine, *MTX* Methotrexate, *CsA* Cyclosporin, *AZA* Azathioprine, *MMF* Mycophenolate mofetil

^**a**^Drugs used for at least one month were analysed

Among the 20 patients who developed subsequent LN, 23.5% of them had heavy proteinuria (urine protein > 3.5 g/day), 9/14 (64.3%) of them had documented hypoalbuminemia (serum albumin < 3.5 mg/dL), and 10% of them had an eGFR < 60 mL/min/1.73m^2^ at their LN diagnosis. Four patients had a decline in renal function along with their LN progression in later years. We analyzed the differences in basal clinical features and lab data between groups based on their presence of heavy proteinuria, hypoalbuminemia, or eGFR < 60 mL/min/1.73m^2^ at LN diagnosis. We only found that patients with eGFR < 60 at LN diagnosis had a significantly lower mean C4 during follow-ups (3.83mg/dL, IQR = 0.80 vs. 10.6mg/dL, IQR = 2.73, *p* = 0.012) and a shortened duration between their TD and LN(1.79 years, IQR = 0.17 vs. 4.82 years, IQR = 1.99, *p* = 0.047). We did not perform further regression models due to the small sample size.

Regarding medications, every patient received HCQ except one in Group 1 with glucose-6-phosphate dehydrogenase deficiency. There was no difference in the use of DMARDs between the two groups. The accumulated corticosteroid dosage was higher in Group 1 than in Group 2, although this result did not show a significant difference statistically (6.2 mg/day vs. 5.1 mg/day, *p* = 0.085).

### Risk factors associated with subsequent LN in jSLE patients

Table 3 shows the Cox proportional hazard model results for predicting factors of subsequent LN. In univariate analysis, the factors significantly associated with an increased risk of subsequent LN were higher average anti-dsDNA antibody and ESR levels, and higher accumulated corticosteroid dose. Complement levels, which are indicators of SLE activity, were inversely associated with subsequent LN development. Higher accumulated corticosteroid dose was associated with subsequent LN, but lost it significance in multivariable analysis. In the multivariable analysis, increased average anti-dsDNA antibody and ESR levels remained significant risk factors for subsequent LN. With every 100 IU/ml increment in the average level of anti-dsDNA antibody, those patients with jSLE were at a higher risk of subsequent LN than their counterparts (HR = 1.29, 95% CI = 1.06–1.57, *p* = 0.013).

**Table 3:** Univariate and multivariate Cox regression analyses of subsequent LN

Variables	**Univariate Model**		**Adjusted Model**	
	HR (95% CI)	*p*-value	HR (95% CI)	*p*-value
Female	2.31 (0.51–10.38)	0.276		
Age at SLE diagnosis (yrs)	0.97 (0.82–1.14)	0.692		
**Clinical manifestations (at SLE diagnosis)**				
Malar rash	0.90 (0.37–2.22)	0.821		
Discoid rash	0.15 (0.02–1.09)	0.061		
Photosensitivity	0.95 (0.34–2.65)	0.926		
Oral ulcer	1.64 (0.66–4.08)	0.290		
Arthritis	1.52 (0.58–3.98)	0.393		
Serositis	1.98 (0.71–5.50)	0.193		
CNS involvement	1.61 (0.21–12.44)	0.651		
Haematology	1.12 (0.43–2.93)	0.818		
Hepatitis	0.56 (0.15–2.06)	0.377		
Leukopenia^a^	0.91 (0.25–3.45)	0.930		
Lymphopenia^b^	1.67 (0.54–5.14)	0.373		
Thrombocytopenia^c^	1.26 (0.35–4.60)	0.726		
**During the period of follow up**				
dRVVT ever positive	1.30 (0.48–3.55)	0.604		
Average (anti-dsDNA antibody/100) (IU/ml)	1.37 (1.16–1.63)	** < 0.001**	1.289 (1.06–1.57)	**0.013**
Average C3 (mg/dL)	0.95 (0.92–0.98)	** < 0.001**	0.971 (0.93–1.01)	0.145
Average C4 (mg/dL)	0.89 (0.80–0.99)	**0.029**	0.972 (0.86–1.10)	0.663
Average ESR (mm/hr)	1.02 (1.01–1.04)	**0.011**	1.022 (1.00–1.04)	**0.045**
Accumulated steroid dose (mg/day)	1.15 (1.05–1.26)	**0.004**	1.001 (0.88–1.14)	0.985
HCQ	1.0 (0–911,390.05)	1		
CsA	1.33 (0.53–3.34)	0.545		
MTX	2.27 (0.75–6.88)	0.147		
MMF	1.06 (0.43–2.61)	0.901		
AZA	0.94 (0.39–2.27)	0.891		

*ESR* Erythrocyte sedimentation rate, *HCQ* Hydroxychloroquine, *MTX* Methotrexate, *CsA* Cyclosporin, *AZA* Azathioprine, *MMF* Mycophenolate mofetil

^a^Leukopenia was defined as white blood cell count < 4000/ µL

^b^Lymphopenia was defined as absolute lymphocyte count < 1500/ µL

^c^Thrombocytopenia was defined as Platelet count < 100,000/ µL

### Effect of anti-dsDNA Ab level on subsequent LN

**Fig. 2:**
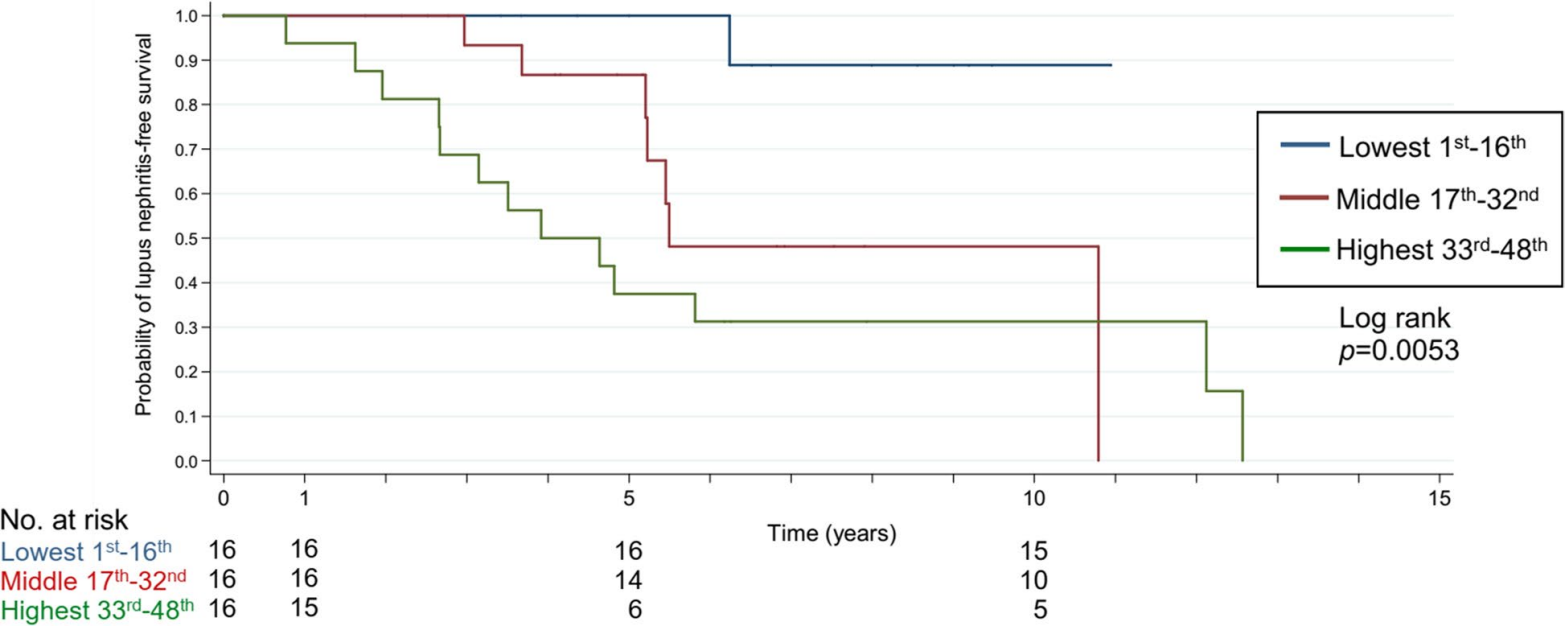
Kaplan–Meier survival curves by SLE diagnosis to developing subsequent lupus nephritis in jSLE populations with different ranks of average anti-dsDNA Ab levels

Because the anti-dsDNA antibody stood out the most in all variables, we performed further analysis to investigate its influence on subsequent LN in patients with jSLE. We ranked the average levels of anti-dsDNA antibody in all 48 jSLE patients from the lowest to the highest and divided them into three equal-sized subgroups. Each subgroup included 16 patients (Supplementary Table 2). The mean anti-dsDNA antibody levels in the subgroups were 237.6 ± 98.1, 542.2 ± 122.7, and 960.9 ± 137.6 IU/ml respectively. In the three subgroups, there were 1 (6.3%), 7 (43.8%), and 13 (81.3%) patients who developed subsequent LN, respectively. Figure 2 shows Kaplan–Meier curves demonstrating the probability of LN-free survival in the subgroups with three ranks of average anti-dsDNA antibody levels during follow-up. The graph shows that patients with the lowest average anti-dsDNA antibody levels had significantly lower renal involvement than the other two groups. The middle subgroup had a hazard ratio of 8.33 to develop subsequent LN compared with the lowest subgroup (95% CI = 1.01–68.48,* p* = 0.049). The highest 33rd–48th subgroup had a hazard ratio of 16.7 for subsequent LN compared with the lowest subgroup (95% CI = 2.16–128.71,* p* = 0.007).

## Discussion

LN remains an essential manifestation of jSLE and plays a role in the long-term prognosis and complications. In Korean and recent Japanese studies, subsequent LN was associated with poorer prognosis and treatment outcomes than early LN [15, 16]. This study found that elevated average anti-dsDNA antibody, low average serum complements, and high average ESR levels were associated with subsequent LN in patients with jSLE. After adjustment, high average anti-dsDNA antibody and ESR levels remained key factors for subsequent LN during serial disease follow-ups.

Studies of patients with SLE reported that 50% to 80% of patients were affected by LN during the disease course [19, 21–25, 32]. Among 103 patients with jSLE in our study, 75 cases (72.8%) had ever developed LN. The variation in LN prevalence in different jSLE cohorts may be due to patient ethnicity, different methods used for LN diagnosis, and length of follow-up period. Studies concerning subsequent renal involvement in patients with SLE were mainly conducted in patients with aSLE. According to different definitions, subsequent LN accounts for 20% to 40% of all SLE patients with LN [13–16]. In our data, 55 patients (53.4%) had evident renal involvement at TD or within one month after TD, and the other 20 patients, 19.4% (20/103) of all jSLE, further developed LN.

Several studies focused on determining the risk factors for delay-developed LN or incident proteinuria, which was used as a surrogate for LN. Most of the research targeted patients with aSLE, and the results varied in different studies due to different definitions. Our study focused on patients with jSLE and defined those with definite LN during follow-up as having subsequent LN instead of incident proteinuria only, which may represent a more profound renal involvement in SLE. Unlike most previous studies, we compared not only the initial manifestations and laboratory data at TD but also the levels of serum serologic markers during a follow-up period.

The status of anti-dsDNA antibody at TD or case enrolment was the most frequently mentioned. Anti-dsDNA IgG, classified as one of the nephritogenic autoantibodies, was widely discussed in earlier studies [33] and is believed to play a role in the development of LN. Previous studies focused on mechanism elucidation suggested that anti-dsDNA antibody may contribute to LN development through direct or non-direct binding to chromatin materials or cross-reactive antigens; however, these results are mainly based on murine models or in vitro studies [34–38]. Reports have indicated that an elevation of anti-dsDNA antibody levels is often observed prior to SLE flares [39, 40] but decreases during lupus flares, including renal flares [39, 41], which may be explained by the deposition of anti-dsDNA immune complexes in tissues. In previous studies, initial positive anti-dsDNA antibody were related to subsequent LN or incident proteinuria [7, 42–45]. The Korean data revealed that aSLE patients developing subsequent LN had a higher anti-dsDNA antibody titre at the TD with an adjusted hazard ratio of 1.004 (95% CI: 1.000–1.007, *p* = 0.026) [45]. In our study, there was no difference in the positivity of anti-dsDNA antibodies at the TD between the two groups. The higher positivity rate of anti-dsDNA antibodies in jSLE than in aSLE, regardless of proteinuria, may explain this discrepancy [46, 47]. However, when we compared the average levels of anti-dsDNA antibody during the following periods, higher mean anti-dsDNA antibody titres correlated strongly with subsequent LN development. For jSLE patients with higher average anti-dsDNA antibody levels, the risk of subsequent LN increased by a 1.29-fold scale fashion every time with an increment of 100 IU/ml of anti-dsDNA antibody. Although the examination kits may vary in different hospitals, and our unit for anti-dsDNA antibody presentation is not universal, the data can be shifted to other universal units using the manufacturer's equation. The hazard ratio increased significantly, primarily in patients with strongly positive anti-dsDNA antibodies. This caused a 5-year LN-free survival of 37.5% after SLE diagnosis, compared with the 100% survival rate in those with nearly negative anti-dsDNA antibody during follow-ups. The findings indicated that persistently or fluctuating high anti-dsDNA antibody levels during clinical follow-ups were important signs of subsequent LN.

In our study, patients with jSLE with a lower average complement level, both C3 and C4, tended to have a higher risk of subsequent LN in the long-term follow-up. A Korean study for patients with aSLE [45] and the UK JSLE Cohort Study for jSLE [32] both reported that a lower C3 level at TD was a risk factor for developing subsequent LN. Lower complement levels have also been associated with increased renal disease activity [48] and renal flares [41] in patients with LN. The core of LN development lies in the intra-renal immune complex deposition that causes complement activation, inflammation, and further kidney damage [49].The alternative complement pathway was also implicated by previous studies that it plays a crucial role in complement-mediated damage in LN [50, 51]. Persistent low C3 levels in our patients with subsequent LN may reflect activation of the alternative complement pathway in the kidneys. Differences in C3 or C4 levels at TD between the two groups were not noted in our study as other studies did, which may be due to our patients’ active disease status at TD. Several studies have also pointed out that patients with jSLE have more episodes of complement level decline and a higher percentage of low C3 compared with patients with aSLE, regardless of the presence of LN [20, 23, 32, 52, 53].

ESR, an inflammation marker, is one of the indices evaluated in the Systemic Lupus Activity Measure (SLAM). Elevated ESR is associated with renal [54] and overall disease activity in SLE [54, 55]. We found that an elevated ESR was related to subsequent LN, and the effect remained after adjusting for other factors. None of the other studies on subsequent LN reported the role of ESR, but active disease status, including higher ACR scores at TD, or higher SLEDAI scores at TD, has been reported to be a risk factor for subsequent LN in patients with jSLE in previous studies [21, 32]. Although we lacked serial data on disease activity in our cases, we found that patients with subsequent LN received a higher accumulated steroid dosage before LN onset than the other group. The effect diminished after adjustment for other factors related to disease activity, such as complement levels and anti-dsDNA antibody titres, indicating higher disease activity and the requirement for higher steroid dosage in Group 1 patients. For other medications, previous studies revealed that antimalarial use was associated with a lower rate of LN [5, 56, 57]. Our data showed no correlation between the other treatments and subsequent LN. However, nearly all our patients using antimalarial for disease control unless contraindicated that we were unable to evaluate the protective effect of using antimalarial as previous studies mentioned.

Regarding the skin manifestations at TD, we found that those with discoid rashes were less likely to have subsequent LN overall. Several studies have reported that patients with SLE with mucocutaneous lesions tend to have less vital organ involvement [58, 59]. A British study that included 241 patients with jSLE found that those without skin manifestations tended to have more haematological and renal involvement [60]. Another cohort of 47 jSLE patients in the U.S. also pointed out the inverse trend of the mucocutaneous lesions and renal involvement in patients with jSLE (OR: 0.5, 95% CI: 0.2–0.9) [22]. Our findings are consistent with those of the above studies. However, the mechanism and relationship of this inverse correlation between discoid rash and LN requires further research.

Our study had several limitations. First, we had a relatively small sample size due to the restriction of the juvenile-onset population, which is generally one-tenth of aSLE [47]. Second, based on the retrospective data collection design, we lacked the serial disease activity score and objective assessment for drug adherence in our study, and there were some incomplete data, such as the status of autoantibodies other than anti-dsDNA and anti-Sm antibodies. The relationship between subsequent LN or proteinuria and other autoantibodies, such as anti-Sm [44, 61], anti-histone [62], anti-RNP [44], and anti-cardiolipin antibodies [10, 43], have also been reported in a few studies. Establishing a relationship between these autoantibodies and subsequent LN development in jSLE may require more comprehensive data. Third, because of a lack of available nephrologists or refusal by the family, only a small portion of our LN cases received renal biopsy, which is currently the gold standard for LN diagnosis. To make a firm LN diagnosis, we used a relatively rigid definition of LN for patients without renal biopsy, which included persistent proteinuria plus the presence of RBC or WBC cellular casts. Last, the patients with jSLE in our study were observed for various durations. Those who remained free from LN may have had subsequent LN if we extended the observing periods. To minimise this possibility, all patients in Group 2 had regular follow-up records for more than one year, and the shortest follow-up duration was 1.65 years. In addition, the mean follow-up durations of the two groups were similar.

Previous studies that searched for risk factors of subsequent LN checked the clinical features or serologic biomarkers at a single period; nevertheless, they did not further consider the change in these markers in the follow-ups. Our study is novel in that we focused on patients with jSLE and collected data not only from TD but also from a full-length follow-up period. To understand the course and prevent the subsequent LN more comprehensively, we still require further investigations involving the dynamic change in disease activity of SLE and other more sensitive biomarkers for early LN detection.

## Conclusion

This study shows that higher average anti-dsDNA antibody and ESR levels during follow-ups are risk factors for subsequent LN among patients with jSLE. These results highlight the importance of close monitoring of renal status in patients with jSLE without renal involvement at TD, especially in those with persistently high anti-dsDNA antibody titres or ESR levels. By early detection of subsequent LN in these young patients at higher risk, we can improve their outcomes in the future.

## Supplementary Information

**Additional file 1: Supplementary Table 1.** The initial lab data of patients with jSLE at the time of diagnosis.

**Additional file 2: Supplementary Table 2.** The list of jSLE patients ranked in order by their average anti-dsDNA antibody levels.

## Data Availability

The datasets used and analysed during the current study are available from the corresponding author on reasonable request.

## Abbreviations

LN: Lupus nephritis
SLE: Systemic lupus erythematosus
jSLE: Juvenile-onset systemic lupus erythematosus
aSLE: adult-onset systemic lupus erythematosus
dsDNA: Double-stranded DNA
ESR: Erythrocyte sedimentation rate
TD: Time of SLE diagnosis
C3: Complement component 3
C4: Complement component 4
ELISA: Enzyme-linked immunosorbent assay
IgG: Immunoglobulin G
DMARDs: Disease-modifying anti-rheumatic drugs
HCQ: Hydroxychloroquine
MTX: Methotrexate
CsA: Cyclosporin
AZA: Azathioprine
MMF: Mycophenolate mofetil
eGFR: Estimated glomerular filtration rate
