# Supplementary material for: Risk factors for subsequent lupus nephritis in patients with juvenile-onset systemic lupus erythematosus: a retrospective cohort study
Source: Pediatr Rheumatol Online J. 2023 Mar 24;21:28. doi: 10.1186/s12969-023-00806-x (PMC10039593; doi:10.1186/s12969-023-00806-x)
Supplement: Supplementary file 1 — Additional file 1: Supplementary Table 1. The initial lab data of patients with jSLE at the time of diagnosis. [file 12969_2023_806_MOESM1_ESM.docx]

**Supplementary Table 1.** The initial lab data of patients with jSLE at the time of diagnosis.

|  | Group 1 (N=20) | Group 2 (N=28) | P value |
| --- | --- | --- | --- |
| C3, median (range) | 66.9 (25.1-151.0) | 63.25(14.4-174.0) | 0.808 |
| C4, median (range) | 9.2 (1.6-39.2) | 7.1 (1.54-34.5) | 0.508 |
| ESR, median (range) | 58.0 (8.0-140.0) (1 missing data) | 37.0 (7.0-140.0)  (2 missing data) | 0.298 |
| Positivity of Anti-dsDNA antibody, n (%) | 14 (87.5%)  (4 missing data) | 21 (80.8%)  (2 missing data) | 0.690 |

jSLE: juvenile-onset systemic lupus erythematosus; C3: complement component 3; C4: complement component 4: dsDNA: double-stranded DNA
