# Supplementary material for: Risk factors for subsequent lupus nephritis in patients with juvenile-onset systemic lupus erythematosus: a retrospective cohort study
Source: Pediatr Rheumatol Online J. 2023 Mar 24;21:28. doi: 10.1186/s12969-023-00806-x (PMC10039593; doi:10.1186/s12969-023-00806-x)
Supplement: Supplementary file 2 — Additional file 2: Supplementary Table 2. The list of jSLE patients ranked in order by their average anti-dsDNA antibody levels. [file 12969_2023_806_MOESM2_ESM.docx]

**Supplementary Table 2.** The list of jSLE patients ranked in order by their average anti-dsDNA antibody levels.

| Rank | Patient No.* | Average anti-dsDNA antibody  (IU/mL) |
| --- | --- | --- |
| 1 | a2 | 29 |
| 2 | a18 | 120 |
| 3 | a16 | 121 |
| 4 | a6 | 150 |
| 5 | **p14** | 199 |
| 6 | a4 | 230 |
| 7 | a24 | 236 |
| 8 | a28 | 244 |
| 9 | a17 | 249 |
| 10 | a11 | 251 |
| 11 | a13 | 273 |
| 12 | a22 | 287 |
| 13 | a27 | 306 |
| 14 | a14 | 318 |
| 15 | a19 | 385 |
| 16 | a25 | 403 |
| 17 | **p15** | 411.03 |
| 18 | a3 | 415.96 |
| 19 | **p2** | 416.03 |
| 20 | **p1** | 417.32 |
| 21 | **p11** | 424.38 |
| 22 | a23 | 431.46 |
| 23 | a15 | 444.32 |
| 24 | **p3** | 528.65 |
| 25 | a10 | 533.71 |
| 26 | a1 | 582.14 |
| 27 | a26 | 607.83 |
| 28 | a12 | 642.67 |
| 29 | **p13** | 655.87 |
| 30 | **p12** | 717.5 |
| 31 | a8 | 717.8 |
| 32 | a9 | 728.68 |
| 33 | a20 | 763.16 |
| 34 | **p17** | 778.65 |
| 35 | a7 | 782.42 |
| 36 | **p8** | 809.57 |
| 37 | **p20** | 870.05 |
| 38 | a21 | 906.36 |
| 39 | **p19** | 952.69 |
| 40 | **p7** | 957.12 |
| 41 | **p16** | 974.52 |
| 42 | **p9** | 976.88 |
| 43 | a5 | 1012.56 |
| 44 | **p10** | 1036.63 |
| 45 | **p5** | 1081.45 |
| 46 | **p6** | 1099.67 |
| 47 | **p18** | 1161.37 |
| 48 | **p4** | 1211.85 |

*Patients with p-(number) were those with subsequent LN (Group 1), while a-(number) were patients without subsequent LN (Group 2).

jSLE: juvenile-onset systemic lupus erythematosus; dsDNA: double-stranded DNA
